# Stability and Fermentability of Green Tea Flavonols in In-Vitro-Simulated Gastrointestinal Digestion and Human Fecal Fermentation

**DOI:** 10.3390/ijms20235890

**Published:** 2019-11-24

**Authors:** Chan-Su Rha, Hyunbin Seong, Young Sung Jung, Davin Jang, Jun-Gu Kwak, Dae-Ok Kim, Nam Soo Han

**Affiliations:** 1 Vitalbeautie Research Division, Amorepacific R&D Center, Yongin 17074, Korea; teaman@amorepacific.com; 2 Brain Korea 21 Center for Bio-Resource Development, Division of Animal, Horticultural, and Food Sciences, Chungbuk National University, Cheongju 28644, Korea; adsm06@naver.com (H.S.); kwakjg93@naver.com (J.-G.K.); 3 Department of Food Science and Biotechnology, Kyung Hee University, Yongin 17104, Korea; chembio@khu.ac.kr; 4 Graduate School of Biotechnology, Kyung Hee University, Yongin 17104, Korea; davin1031@khu.ac.kr

**Keywords:** bioaccessibility, flavonol, green tea, in vitro digestion, in vitro fermentation, short-chain fatty acid

## Abstract

Flavonols, the second most abundant flavonoids in green tea, exist mainly in the form of glycosides. Flavonols are known to have a variety of beneficial health effects; however, limited information is available on their fate in the digestive system. We investigated the digestive stability of flavonol aglycones and glycosides from green tea under simulated digestion and anaerobic human fecal fermentation. Green tea fractions rich in flavonol glycosides and aglycones, termed flavonol-glycoside-rich fraction (FLG) and flavonol-aglycone-rich fraction (FLA) hereafter, were obtained after treatment with cellulase and tannase, respectively. Kaempferol and its glycosides were found to be more stable in simulated gastric and intestinal fluids than the derivatives of quercetin and myricetin. Anaerobic human fecal fermentation with FLG and FLA increased the populations of *Lactobacilli* spp. and *Bifidobacteria* spp. and generated various organic acids, such as acetate, butyrate, propionate, and lactate, among which butyrate was produced in the highest amount. Our findings indicate that some stable polyphenols have higher bioaccessibilities in the gastrointestinal tract and that their health-modulating effects result from their interactions with microbes in the gut.

## 1. Introduction

Green tea is the most commonly consumed beverage in the world and contains a wide range of bioactive compounds, including caffeine and flavan-3-ols (catechins) [1,2]. Green tea leaves consist of about 30% polyphenols based on dry weight, of which ≈15% are flavan-3-ols, and less than 1% are flavonols and flavones [3,4]. Many studies on the beneficial health effects of green tea, mainly of flavan-3-ols, have been published [1,5]. The flavonols and flavones in tea are mainly found as glycosides, and their types and compositions vary with tea type and cultivar [2,3]. Aglycones of flavonols such as kaempferol, myricetin, and quercetin as well as their glycosides have comparable or higher antioxidant capacities than that of vitamin C [6]. Moreover, flavonols provide health benefits such as anticancer [7] and antihyperlipidemic effects [8], and flavonol intake improves a variety of biomarkers of cardiovascular disease risk, such as total cholesterol, low-density lipoprotein, and triacylglycerol levels [9]. Furthermore, the bioavailability of flavan-3-ols can be modulated by other phenolics such as genistein, luteolin, and quercetin [10]. Flavan-3-ol bioavailability decreases after methylation by catechol-*O*-methyltransferase in the body [11]. Accordingly, quercetin has been reported to increase the uptake of flavan-3-ols in models in vitro and in vivo, partly due to its inhibition of catechol-*O*-methyltransferase [12].

Flavonol aglycones contain different glycosidic bonds. Furthermore, polyphenolic structures and compositions vary by plant source, influencing the bioaccessibility and bioavailability of flavonol glycosides [13,14]. Attaching the same sugar at different positions of a quercetin aglycone does not affect the bioavailabilities of the resulting glycosides in humans [15]. However, the bioavailability of quercetin diglycoside in humans is lower than that of quercetin monoglycoside [14], implying that more complex flavonol glycosides are less hydrolyzed under digestive conditions. Quercetin glycosides are efficiently hydrolyzed to quercetin by β-glucosidases in the small intestine [16]. Quercetin 4′-glycoside is catabolized through ring fission by intestinal bacteria into phenolic acids, such as 3,4-dihydroxyphenylacetic acid, which has higher biological activities, such as antioxidant effects, than its parent molecule [17]. Furthermore, it has been reported that a relatively large portion of ingested rutin remains intact in the ileal fluid and that rutin metabolism occurs mainly via microbial biotransformation in the large intestine [18].

Many studies on the fates of quercetin, isoquercitrin, and rutin as common flavonol aglycones and glycosides by microbial degradation have been reported [19,20]. Furthermore, Simons et al. [21] measured the degradation rate of some flavonoids fermented in vitro with human feces, but none of the flavonol glycosides were considered. Moreover, the metabolic fates of flavonoids under the actions of various microbes in the human microbiota working on C-ring fission of dietary flavonoids have been studied [22]. However, information on the changes during digestion of green tea flavonol glycosides having various glycosidic linkages remains limited.

Therefore, we investigated the digestive stabilities of the flavonol-glycoside-rich fraction (FLG) and flavonol-aglycone-rich fraction (FLA) of green tea extracts in various simulated gastrointestinal fluids. We also evaluated the changes in the microbial populations and short-chain fatty acid (SCFA) profile upon anaerobic human fecal fermentation in vitro with FLG and FLA. Identification and quantification of flavonols in FLG and FLA under different digestive conditions were performed using high-performance liquid chromatography (HPLC).

## 2. Results and Discussion

### 2.1. Stabilities of Kaempferol, Myricetin, and Quercetin in In Vitro Digestion

Three flavonol aglycones (kaempferol, myricetin, and quercetin) were subjected to four simulated digestive environments—one gastric and three intestinal (Table 1). Kaempferol shows no significant (*p* > 0.05) changes in the simulated gastric fluid (SGF), but significant (*p* < 0.05) changes in the simulated intestinal fluid (SIF) conditions used in this study (Table 1). Kaempferol is more stable than the two others in the SIF conditions. Myricetin exhibits no significant (*p* > 0.05) changes under SGF conditions but undergoes complete degradation upon digestion in pancreatin, brush border membrane vesicles (BBMVs), and their co-treatment mixture (Table 1). Quercetin shows no significant (*p* > 0.05) differences in relative stability under the SGF conditions, and its complete degradation upon co-treatment with pancreatin and BBMV is observed (Table 1).

Similar to the findings for myricetin in this study, a previous study revealed that myricetin degradation is more influenced by neutral pH intestinal digestive conditions than by acidic SGF [23]. For that study, the half-life of myricetin was reported to be approximately 2.11 h at pH 6.8, whereas its half-life at pH 7.4 was too low to be measured owing to its rapid degradation [24]. Furthermore, myricetin has been reported to be most stable at pH 2.0 and exhibits higher solubility at pH 1.2–3.0 than at pH 6.8–7.4 [24]. Similarly, quercetin has been reported to undergo extensive degradation as pH increases [25]. Therefore, it may be suggested that, although myricetin and quercetin are stable under gastric conditions, they undergo faster degradation when passing through the intestine, which leads to their low bioaccessibility in the body. Kaempferol has been previously reported to be stable in simulated digestive conditions [26]. The resistance to degradation in SIF observed for kaempferol in the present study indicates that it is absorbed intact into systemic circulation during digestion in the gastrointestinal tract and serves as a crucial bioactive phenolic compound in the body.

### 2.2. Gastrointestinal Stabilities of FLG and FLA in In Vitro Digestion

**Figure 1:**
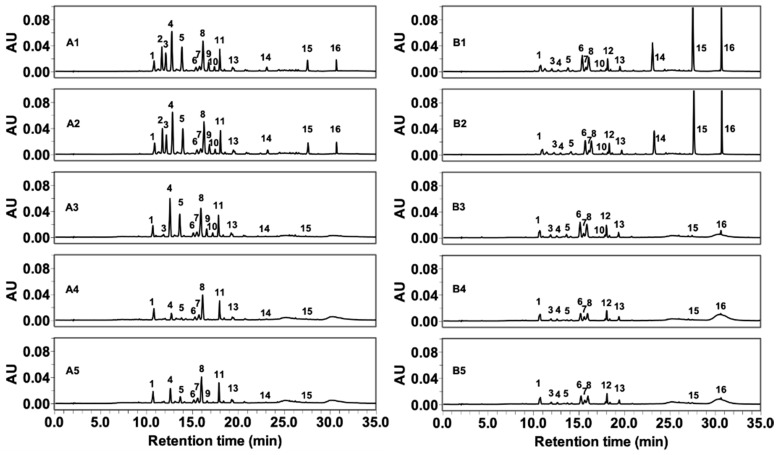
High-performance liquid chromatography (HPLC) traces after (**A**) FLG and (**B**) FLA in in vitro digestion (600 µg/mL) at wavelength of 365 nm. The number after A and B on the HPLC traces indicates the digestive conditions: 1, control; 2, gastric; 3, pancreatin; 4, brush border membrane vesicle (BBMV); 5, pancreatin and BBMV combined. The identification of numbered peaks is presented in Table 2.

The digestive stability of these flavonol glycosides and aglycones in more complex mixtures, namely FLG and FLA from green tea, were investigated. The content of kaempferol, myricetin, and quercetin was 5.91, 4.15, and 7.72 mg/g for FLG and 34.71, 15.94, and 38.21 mg/g for FLA, respectively, in our previous research [27]. The phenolic compounds identified in FLG and FLA are summarized in Table 2. The digestive changes of flavonol glycosides and aglycones in FLG and FLA are depicted in Figure 1 with numbered peaks (1–16). The 16 phenolic glycosides and aglycones were identified in our previous high-resolution mass analysis study [27].

**Figure 2:**
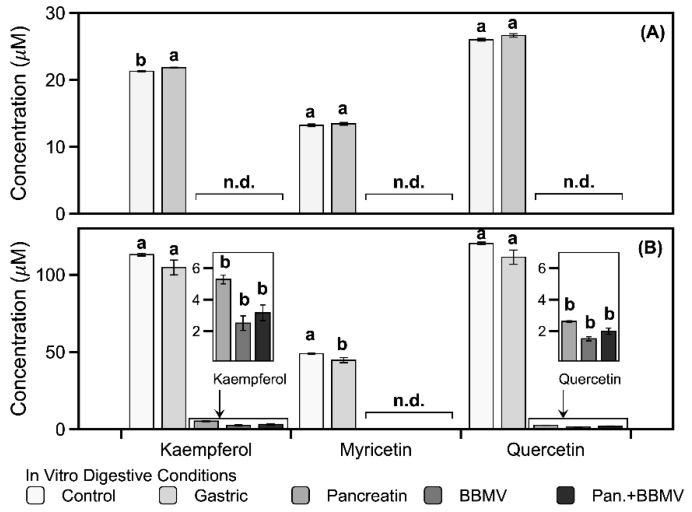
Changes of flavonol aglycones (kaempferol (16), myricetin (14), and quercetin (15)) in (**A**) FLG and (**B**) FLA after in vitro digestion. Different lowercase letters on the bars of each aglycone represent significant differences according to the Tukey–Kramer honest significant difference test (*p* < 0.05). Pan.+BBMV: co-treatment with pancreatin and BBMV. n.d.: not detected.

Much like the results obtained for the digestive stabilities of the pure flavonol aglycones (Table 1), the flavonol aglycones in FLG and FLA are stable under SGF conditions but unstable under SIF conditions (Figure 2). None of the flavonol aglycones in FLG (or myricetin (14) in FLA) are detected under intestinal conditions (Figure 2). In FLA treated with pancreatin, BBMV, and a mixture of pancreatin and BBMV, kaempferol (16) is degraded by 95.3%, 97.8%, and 97.2%, respectively, whereas quercetin (15) is degraded by 97.8%, 98.7%, and 98.3% (Figure 2B). These results indicate that the flavonols in FLG and FLA are unstable and exhibit poor bioaccessibility under intestinal conditions at a pH of 7.0.

**Figure 3:**
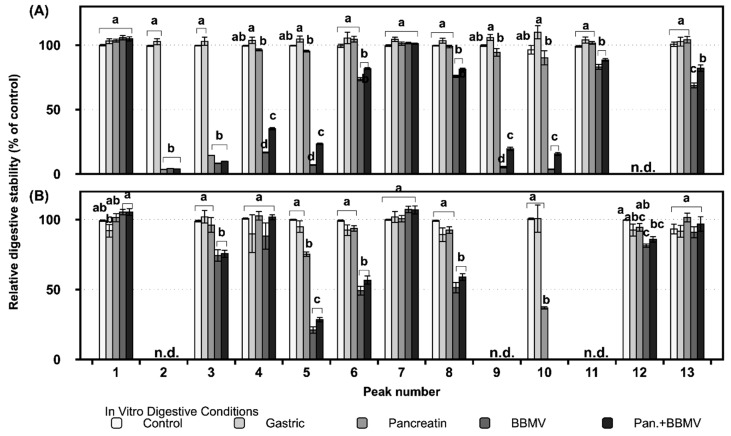
Changes of glycosides in (**A**) FLG and (**B**) FLA after in vitro digestion. Different lowercase letters on the bars of each compound (peak number) represent significant differences according to the Tukey–Kramer honest significant difference test (*p* < 0.05). Pan.+BBMV: co-treatment with pancreatin and BBMV. n.d.: not detected. Refer to the peak number in Figure 1.

Most of the glycosides in FLG are stable under gastric conditions, but some quercetin and myricetin glycosides, such as those represented by peaks 2, 3, 4, 5, 9, and 10, are degraded upon treatment with pancreatin, BBMV, and a combination of both (Figure 3A). Among the 16 phenolics detected in FLG and FLA, kaempferol-3-*O*-rhamnosylgalactoside (12) is not detected in FLG after digestion in vitro (Figure 3A), due to the fact that it is not present in the original FLG (Figure 1A). Furthermore, since myricetin-3-*O*-galactoside (2), quercetin-3-*O*-galactoside (9), and kaempferol-3-*O*-glucosylrutinoside (11) are not found in FLA prior to in vitro digestion (Figure 1B), they are not detected in FLG after digestion either (Figure 3B). Apigenin-6-*C*-glucosyl-8-*C*-arabinoside (1) and quercetin-3-*O*-rhamnosylglucoside (7) in FLG are the most stable of the flavonoids under all four digestive conditions (Figure 3A), while apigenin-6-*C*-glucosyl-8-*C*-arabinoside (1), quercetin-3-*O*-galactosylrutinoside (4), quercetin-3-*O*-rhamnosylglucoside (7), and kaempferol-3-*O*-rhamnosylglucoside (13) in FLA show no significant (*p* > 0.05) degradation compared with that of the control (Figure 3B).

The glycosides in FLA are more stable under all the digestive conditions than those in FLG (Figure 3), indicating that the matrix surrounding the flavonoids affects their degradation in the digestive tract. The flavonoids common to both FLG and FLA that exhibit over 50% digestive stability are apigenin-6-*C*-glucosyl-8-*C*-arabinoside (1), quercetin-3-*O*-rhamnosylglucoside (7), apigenin-6-*C*-glucoside (8), and kaempferol-3-*O*-rhamnosylglucoside (13) (Figure 3).

Flavone *C*-monoglycosides have been observed to be resistant to degradation by chemical/biological methods and gut digestion conditions [28]. Flavone *C*-glycosides such as apigenin-6-*C*-glucosyl-8-*C*-arabinoside (1) may be absorbed in the intact form in the intestine [28]. A small fraction of apigenin-*C*-glycosides (1 and 8) in FLG had their glycosidic linkages cleaved by intestinal enzymes (data not shown), indicating that most *C*-glycosides of flavones migrate from the small intestine to the large intestine without degradation. Kaempferol and its glycosides are more stable than myricetin, quercetin, and their glycosides (Figure 2 and Figure 3) [29]. Therefore, kaempferol and its glycosides may be more available for potential absorption into the body and transport to the large intestine.

Flavonol glycosides show greater degradation upon exposure to BBMV only and a mixture of pancreatin and BBMV than when exposed to pancreatin only (Figure 3), implying that BBMV plays a vital role in flavonoid digestion. BBMV contains various enzymes in the small intestine microvilli. Lactase-phlorizin hydrolase (LPH) exists on the apical surfaces of brush border enterocytes, where it is anchored into the membrane by its *C*-terminal end [30]. LPH catalyzes the hydrolysis of a variety of flavonoid β-glucosides, such as phlorizin [30]. Flavonols are degraded by LPH or conjugated with glucuronic acid by uridine 5′-diphospho-glucuronosyltransferase before absorption in the body [31,32]. Treatment of Caco-2 cells with lactase has been reported to increase quercetin bioavailability [31]. The pH changes through stomach to intestine cause degradation of flavonol glycosides, the digestive enzymes hydrolyze the residual flavonol glycosides in the small intestine, and the flavonols that reach the large intestine are finally metabolized by the microbiota. Therefore, the degradation and absorption of polyphenols in green tea depend on intestinal microflora and LPH production, meaning that polyphenol bioavailability will vary between individuals. Moreover, enzymatically treated flavonols, such as FLA in this study, may have higher bioavailability than flavonol glycosides, regardless of LPH in the body.

### 2.3. Modulating Interaction of FLG and FLA on Microbial Populations in In Vitro Fecal Fermentation

**Figure 4:**
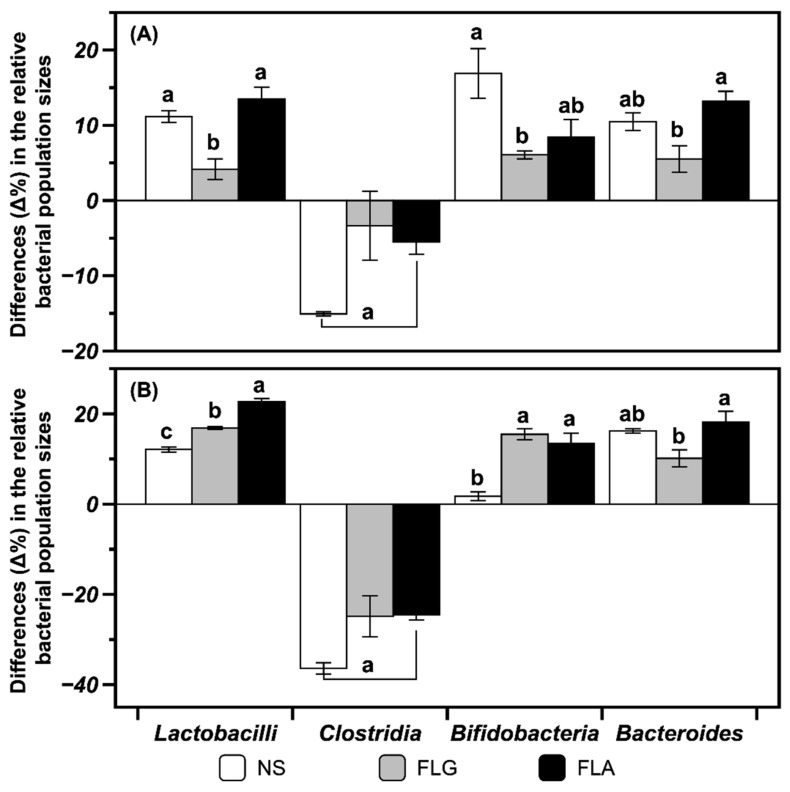
Effects of FLG and FLA on the microbial population in an in vitro fecal fermentation for (**A**) 12 h and (**B**) 24 h. Differences (Δ%) in the relative bacterial population sizes with respect to the total number of bacteria were evaluated after fermentation of FLG and FLA from green tea extract. The following calculation was applied: [(selected bacterial number at 12 or 24 h/total bacteria number at 12 or 24 h) – (selected bacterial number at 0 h/total bacteria number at 0 h)] × 100. NS stands for no substrate as a control. Different lowercase letters on the bars of each microorganism represent significant differences according to the Tukey–Kramer honest significant difference test (*p* < 0.05).

Culture media from in vitro batch fermentations with fecal samples were collected at 12 and 24 h. Quantitative polymerase chain reaction (qPCR) analysis was used to enumerate the population of bacterial groups upon fecal fermentation in vitro. The populations of the lactic acid bacteria *Bifidobacteria* spp. and *Lactobacilli* spp. are significantly (*p* < 0.05) increased upon 24 h fecal fermentation with FLG and FLA compared with those of control not treated with FLG or FLA (Figure 4). The population of a representative pathogenic bacterium, *Clostridium*, in control decreases after fermentation (Figure 4). The fermentations with FLG and FLA also bring about *Clostridium* population similar to that of the control (Figure 4). In general, the FLA-treated fermentation exhibits higher stimulation of bacterial growth than the FLG-treated fermentation. There are no significant (*p* > 0.05) differences in the population of the symbiotic microorganism *Bacteroides* in the FLG- and FLA-added fermentations compared with that of the control (Figure 4). Phenolics are known to be biotransformed in the colonic microflora, which may affect their absorption and biological effects [22]. Some phenolics have been reported to have inhibitory effects on pathogens but to enhance the growth of *Lactobacillus rhamnosus* [33]. Quercetin as one of the flavonols has been reported to have prebiotic effects on *Bifidobacterium adolescentis*, producing nitric-oxide-suppressing agents [34].

### 2.4. Effects of FLG and FLA on SCFAs and Lactate Production in In Vitro Fecal Fermentation

SCFAs are important metabolites for colon health because, in addition to being the primary source of energy for colon cells, they are signaling chemicals that cause anti-inflammatory and anticarcinogenic effects [35,36]. Therefore, the production of SCFAs during in vitro fecal fermentation is considered a unique biomarker for the analysis of prebiotic activity against various substances.

**Figure 5:**
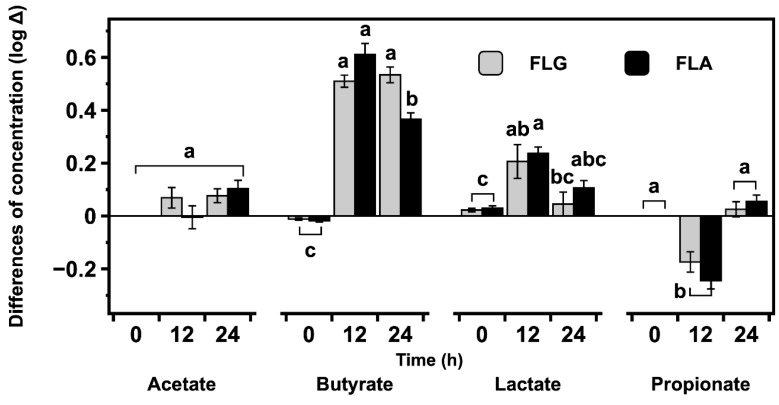
Changes in concentrations of acetate, butyrate, lactate, and propionate during in vitro fecal fermentation with FLG and FLA. The following calculation was applied: [log (acid concentration after fermentation – acid concentration at blank-treated control)]. Different lowercase letters on the bars of each acid represent significant differences according to the Tukey–Kramer honest significant difference test (*p* < 0.05).

The differences of concentrations of lactate and SCFAs (acetate, propionate, and butyrate) produced during fecal fermentation with FLG and FLA are shown in Figure 5. The amounts of lactate and SCFAs produced in in vitro fecal fermentation with FLG and FLA are generally higher than those of the control without FLG or FLA. Acetate production is slightly higher for fecal fermentation with FLG and FLA. The amount of lactate is higher at 12 h and then lower at 24 h compared to the control. The propionate level is lower at 12 h and then recovered to a similar level to the blank-treated control. The butyrate concentration is 16.55 mM after fecal fermentation with FLA at 12 h and 19.17 mM after fermentation with the FLG at 24 h (data not shown).

In the fecal fermentation supplemented with FLG, the conversion of lactate into butyrate may account for the lack of significant (*p* > 0.05) difference between the butyrate concentrations of the 12 and 24 h anaerobic fecal fermentation. Compared with acetate, propionate, and lactate, butyrate is produced in higher amounts from fecal fermentations with FLG and FLA (Figure 5), suggesting that both fractions are utilized as the main substrates of intestinal microorganisms and have beneficial effects on human gut health through the butyrate production.

*Lactobacilli* and *Bifidobacteria* are known to produce lactate and acetate, respectively, as final metabolites [37,38,39]. The final metabolites are not distinctive in *Bacteroides*, that is, different SCFAs and lactate can be produced varying with growth phase, medium composition, and metabolic cross-feeding [40]. In the present study, butyrate is produced in higher amounts than acetate, propionate, and lactate in anaerobic fecal fermentation (Figure 5). In previous studies, butyrate production was observed to increase significantly in rats fed with the lactic acid bacteria *L. rhamnosus* together with malt [41] and, furthermore, lactate and acetate can be converted to butyrate by human gut microbes such as *Bi. adolescentis* [42]. Butyrate has been demonstrated to have beneficial effects on the gut by regulating inflammation and supporting energy metabolism in the microbiota [43], and macrophages treated with butyrate were found to exhibit decreased production of proinflammatory cytokines such as interleukin-6 [44].

Quercetin glycosides such as isoquercitrin and rutin have been reported to be anaerobically deconjugated and degraded into lower molecular weight products such as quercetin and phenolic acid through in vitro human fecal fermentation [45]. The flavonols undergo ring fission and dehydroxylation by gut microbiota in humans [22]. The type of glycosidic bond between the flavonol and sugar moiety influences the degradation rate in the digestive tract, with *C*-glycosidic bonds exhibiting much slower cleavage than *O*-glycosidic bonds [46]. The production of lactate and SCFAs in anaerobic fecal fermentation in this study implies that gut bacteria utilize both flavonol glycosides and aglycones. The results in this study (Figure 4 and Figure 5) suggest that flavonols and other phenolics in the FLG and FLA from green tea are resistant to the digestive conditions in the upper small intestine, pass through the small intestine, and then modulate large intestinal microbial growth and production of SCFAs and lactate to produce health benefits in the body.

## 3. Materials and Methods

### 3.1. Chemicals and Reagents

Kaempferol, myricetin, quercetin, dimethyl sulfoxide (DMSO), MgSO_4_·7H_2_O, bile salts, *l*-cysteine hydrochloride, hemin, resazurin solution, pepsin (from porcine mucosa), pancreatin (from porcine pancreas), and formic acid were purchased from Sigma-Aldrich Co., LLC (St. Louis, MO, USA). HCl, NaCl, K_2_HPO_4_, KH_2_PO_4_, NaHCO_3_, MgCl_2_, KCl, and CaCl_2_·2H_2_O were purchased from Junsei Chemical Co., Ltd. (Tokyo, Japan). Tween 80 was purchased from VWR International, LLC (Radnor, PA, USA). Methanol (HPLC grade) and acetonitrile (HPLC grade) were obtained from Thermo Fisher Scientific (Waltham, MA, USA). Water (HPLC grade) was purchased from Burdick & Jackson (Muskegon, MI, USA). The fresh porcine small intestine was purchased from a local food market. All other chemicals were of analytical grade or higher.

### 3.2. Preparation of FLG and FLA

The FLG and FLA from dried green tea extract were obtained by the method used in our previous studies [27,47]. In brief, fresh green tea was harvested at Osulloc Farm (Jeju-do, Republic of Korea) from May to June 2017. Dried green tea extracts were obtained from a solution prepared with aqueous ethanol (70% (*v/v*)) at 60 °C for 3 h. The FLG and FLA were obtained by enzymatic treatment and preparative HPLC purification.

### 3.3. Preparation of BBMVs

Whole ileum mucosa containing BBMVs was prepared according to the method used by Gnoth et al. [48]. The mucosa was gently scraped from the underlying muscular tissue of porcine small intestine using a glass slide. The mucosa fraction (500 mg) in buffer (2 mM Tris-HCl and 50 mM mannitol; pH 7.1) was mixed using a homogenizer (Ultra-Turrax T25; IKA-Works, Inc., Cincinnati, OH, USA) for 30 s at 24,000 rpm. MgCl_2_ was added to the homogenized mucosa fraction to a final concentration of 10 mM, and the mixture was allowed to stand for 15 min. After the homogenate was centrifuged at 1400 *g* for 12 min, the supernatant was collected and centrifuged at 16,000 *g* for 20 min. The supernatant was discarded, and the pellet was resuspended in phosphate-buffered saline (PBS; pH 7.0) and then homogenized in a loose-fitting glass-Teflon tissue grinder at low speed. After another centrifugation at 16,000 *g* for 20 min, the pellet was resuspended in PBS (100 μL). The efficiency of the BBMV preparation was assessed by measuring the activity of marker enzymes (isomaltase, lactase, maltase, and sucrase), through the glucose liberation during incubation of the BBMV with isomaltose, lactose, maltose, and sucrose, respectively.

### 3.4. In Vitro Digestibility of Pure Flavonols, FLG, and FLA

Three flavonol aglycones (kaempferol, myricetin, and quercetin), FLG, and FLA were used to evaluate in vitro digestibility in four reaction conditions—gastric, pancreatin, BBMV, and co-treatment with pancreatin and BBMV. The last three mimic intestinal conditions. In vitro digestion was accomplished using the modified method indicated by Minekus et al. [49] (Table 3). Pure flavonols (3.0 mg), FLG (3.0 mg), and FLA (3.0 mg) were reacted in 2.5 mL mixed solution with enzyme and buffer. SGF (pH 3.0) consisted of 6.9 mM KCl, 0.9 mM KH_2_PO_4_, 25 mM NaHCO_3_, 47.2 mM NaCl, 0.1 mM MgCl_2_·6H_2_O, 0.5 mM (NH_4_)_2_·CO_3_, and 0.15 mM CaCl_2_·2H_2_O. SIF (pH 7.0) was made up of 6.8 mM KCl, 0.8 mM KH_2_PO_4_, 85 mM NaHCO_3_, 38.4 mM NaCl, 0.33 mM MgCl_2_·6H_2_O, and 0.6 mM CaCl_2_·2H_2_O. Pepsin solution (25,000 units/mL) and pancreatin solution (800 units/mL; as pancreatin α-amylase activity) were prepared in SGF and SIF, respectively. The enzyme reactions were stopped by the addition of stopping solution. The reaction tubes were instantly frozen by immersion in liquid nitrogen and were then stored at −80 °C prior to use. The frozen samples were solubilized by sonication for 20 min and appropriately diluted with 10% (*v/v*) DMSO in methanol for analyses.

### 3.5. In Vitro Fecal Batch Fermentation of FLG and FLA

Fresh feces were collected from seven healthy adult human donors who were confirmed for no administration of antibiotics, prebiotics, or probiotics and no recent history of gastrointestinal disorders. In vitro fecal batch fermentation with FLG and FLA was performed according to the method indicated by Mandalari et al. [50]. For the fermentation, a 135 mL portion of the basal growth medium (2 g/L of peptone water, 1 g/L of yeast extract, 2 g/L of NaHCO_3_, 0.1 g/L of NaCl, 40 mg/L of K_2_HPO_4_, 40 mg/L of KH_2_PO_4_, 10 mg/L of MgSO_4_·7H_2_O, 10 mg/L of CaCl_2_·2H_2_O, 0.5 g/L of bile salts, 0.5 g/L of *l*-cysteine hydrochloride, 50 mg/L of hemin, 10 μL/L of vitamin K_1_, and 2 mL/L of Tween 80) was added to water-jacketed fermenter vessels (300 mL capacity). To obtain a fecal slurry, 10% (*w/v*) of fecal matter in 100 mM PBS (pH 7.0) was homogenized. The resulting slurry (15 mL) and 150 mg FLG or FLA were poured into the water-jacketed fermenter vessels and stirred magnetically. The pH and temperature of the fermenter were automatically maintained at 6.8 and 37 °C, respectively. Throughout the fermentation, oxygen-free nitrogen gas was sparged into the fermenter at a flow rate of 15 mL/min for the maintenance of anaerobic conditions. After 12 and 24 h of fermentation, 5 mL of the reaction media from the vessels were collected for quantitative real-time polymerase chain reaction (qRT-PCR) and SCFA analysis.

### 3.6. Enumeration of Intestinal Bacteria Using qRT-PCR

To enumerate intestinal bacteria, qRT-PCR was conducted using genus-specific primer sets according to the methods previously reported in Seong et al. [51]. The method of extraction, amplification, and detection of purified bacterial DNA was the same as that presented in Section 2.4. in Seong et al. [51]. Furthermore, the relative quantities and ratios of target genes encoding 16S rRNA gene sequences of the bacterial taxa were calculated as the following calculations: *E*^Δ*CT*^ and *E*^Δ*CT* (fermentation sample)^/*E*^Δ*CT* (original bacterial community)^, respectively, where *E* is the efficiency of the primer calculated from the slope of the standard curve (*E* = 10^−1/slope^), and Δ*CT* is the *CT* value of the target bacterial population normalized against the *CT* value of the total bacterial population [52].

### 3.7. Determination of Flavonoids, Lactate, and SCFAs Using HPLC Systems

The flavonoid analysis was performed according to the previous research with minor modifications [27]. Briefly, the flavonoid standards, in vitro digesta of FLG and FLA, and in vitro fermentation samples were dissolved in 10% (*v/v*) DMSO in absolute methanol and filtered through a 0.2 μm regenerated cellulose filter (Sartorius, Göttingen, Germany). Afterward, flavonol content of samples was quantified using Waters Alliance HPLC (Milford, MA, USA) installed with a UV detector and a C_18_ column (Poroshell 120 SB; 4.6 × 150 mm, 120 Å, 2.7 μm; Agilent Technologies, Inc., Santa Clara, CA, USA). The column temperature was maintained at 30 °C. Flavonols were monitored at a wavelength of 365 nm. Five microliters of injection were carried out. The flow rate was 0.8 mL/min. Two mobile phases consisted of 0.1% (*v/v*) formic acid in water (solvent A) and 0.1% (*v/v*) formic acid in acetonitrile (solvent B). Refer to the detailed elution program of our previous study [27].

The amounts of SCFAs and lactate produced in the fecal fermentations were analyzed using a 1260 Infinity HPLC (Agilent Technologies, Inc.) with an Aminex HPX-87H column (300 × 7.8 mm, 9 μm; Bio-Rad Laboratories, Inc., Hercules, CA, USA). The mobile phase was 5 mM H_2_SO_4_ in water. Flow rate, injection volume, and detection wavelength were 0.6 mL/min, 20 µL, and 215 nm, respectively. Amounts of SCFAs and lactate after fermentation were calculated using their standard curves corresponding to concentrations of 1 and 200 mM.

### 3.8. Statistical Analysis

Data are presented as the means ± standard errors of the mean (*n* = 3). One-way analysis of variance was performed using comparisons for all pairs by the Tukey–Kramer honest significant difference test (*p* < 0.05) using JMP 12 for Windows 7 or higher (SAS Institute Inc., Cary, NC, USA). Statistical analysis of the SCFAs, lactate, and qRT-PCR data was performed with SPSS for Windows Version 22 (IBM, Chicago, IL, USA).

### 3.9. Ethical Statement

The experiment using BBMV from slaughtered porcine did not entail any ethical issues as it was obtained as is from a commercial source. The human fecal samples were obtained according to the guidelines of the Division of Animal, Horticultural, and Food Sciences at Chungbuk National University (CBNU; Cheongju, Republic of Korea). The study protocol and consent forms were approved by the Institutional Review Board of CBNU, Republic of Korea (CBNU-201905-BR-839-01).

### 3.10. Data Availability

The datasets generated during and/or analyzed during the current study are available from the corresponding authors on reasonable request.

## 4. Conclusions

Green tea fractions rich in flavonol glycosides and aglycones, termed FLG and FLA, were obtained after tannase and cellulase treatments, respectively. The digestive stabilities of the FLG and FLA were found to depend on the type of aglycone, glycosidic bond, and sugar moiety. Kaempferol glycosides were most stable in in vitro digestive conditions among the flavonols tested in this study. Fermentation with FLG and FLA increased the populations of the lactic acid bacteria *Lactobacilli* spp. and *Bifidobacteria* spp. in anaerobic human fecal fermentation in vitro, during which acetate, butyrate, propionate, and lactate are produced. Thus, the findings in this study indicate that the digestive stability of flavonoids influences their bioaccessibility in the gastrointestinal tract and that the populations of intestinal microflora also affect the bioactivity of flavonoids and their metabolites in the body. Further studies to evaluate the bioavailability of FLG and FLA in animal models in vivo and to study the pharmacodynamic interactions of these fractions and green tea catechins in clinical trials are warranted.

## 5. Patents

The source of FLG and FLA are part of the patent application no. KR10-2019-0054542 entitled “Method for manufacturing green tea extract, and green extract therefrom.”

## Figures and Tables

**Table 1:** The relative stability of flavonol aglycones in four digestive conditions in vitro.

Compound	Digestive Conditions				
	Control	Gastric	Pancreatin	BBMV ^a^	Pancreatin + BBMV ^b^
**Kaempferol**	99.20 ± 4.30 ^Ac^	93.40 ± 2.60 ^A^	65.50 ± 0.50 ^BC^	72.23 ± 0.63 ^B^	54.97 ± 0.58 ^C^
**Myricetin**	101.60 ± 1.53 ^A^	98.97 ± 2.36 ^A^	n.d. ^d^	n.d.	n.d.
**Quercetin**	99.07 ± 5.48 ^A^	96.47 ± 1.35 ^A^	9.23 ± 0.35 ^B^	4.47 ± 0.12 ^C^	n.d.

^a^ BBMV: brush border membrane vesicle. ^b^ Pancreatin + BBMV: co-treatment with pancreatin and BBMV. ^c^ Data are expressed as a percentage (%) compared with control in each condition. Data with different uppercase letters in the same row represent significant differences according to the Tukey–Kramer honest significant difference test (*p* < 0.05) with standard error of the mean. ^d^ n.d. means not detected.

**Table 2:** Identification of phenolic compounds in flavonol-glycoside-rich fraction (FLG) and flavonol-aglycone-rich fraction (FLA).

Peak No. ^a^	Compound ^b^	Peak No.	Compound
1	Apigenin-6-*C*-glucosyl-8-*C*-arabinoside	9	Quercetin-3-*O*-galactoside
2	Myricetin-3-*O*-galactoside	10	Quercetin-3-*O*-glucoside
3	Myricetin-3-*O*-glucoside	11	Kaempferol-3-*O*-glucosylrutinoside
4	Quercetin-3-*O*-galactosylrutinoside	12	Kaempferol-3-*O*-rhamnosylgalactoside
5	Quercetin-3-*O*-glucosylrutinoside	13	Kaempferol-3-*O*-rhamnosylglucoside
6	Quercetin-3-*O*-rhamnosylgalactoside	14	Myricetin
7	Quercetin-3-*O*-rhamnosylglucoside	15	Quercetin
8	Apigenin-6-*C*-glucoside or its isomer	16	Kaempferol

^a^ The corresponding chromatograms are depicted in Figure 1 with numbered peaks. ^b^ Refer to the previous report of Rha et al. [27] for the mass of molecular ions and the fragmentations by high-resolution mass spectrometry.

**Table 3:** Conditions of in vitro digestion of pure flavonols, FLG, and FLA.

Conditions ^a^	Digestive Enzyme	Buffer	Stopping Solution	Reaction Temperature and Time
**Gastric**	pepsin (160 μL)	SGF ^b^ (750 μL), 0.3 M CaCl_2_ (0.5 μL), 1 M HCl (1.0 μL), water (88.5 μL); pH 3.0	solvent 1 ^c^ (400 μL) + water (100 μL)	37 °C, 2 h
**Pancreatin**	pancreatin (250 μL)	SIF ^d^ (550 μL), bile (125 μL), 0.3 M CaCl_2_ (2 μL), 1 M HCl (10 μL), water (63 μL); pH 7.0	solvent 2 ^e^ (400 μL) + water (100 μL)	37 °C, 2 h
**BBMV** ^f^	BBMV (100 μL)	SIF (550 μL), bile (125 μL), 0.3 M CaCl_2_ (2 μL),1 M HCl (10 μL), water (213 μL); pH 7.0	solvent 2 (400 μL) + water (100 μL)	37 °C, 4 h
**Pancreatin + BBMV**	pancreatin (250 μL), BBMV (100 μL)	SIF (550 μL), bile (125 μL), 0.3 M CaCl_2_ (2 μL),1 M HCl (10 μL), water (63 μL); pH 7.0	solvent 2 (400 μL)	37 °C, 2 h or 37 °C, 4 h

^a^ Three milligrams of FLG or FLA dissolved in 1 mL of water were used to evaluate digestive stability. The total reaction volume in each condition was 2.5 mL. ^b^ SGF: simulated gastric fluid. ^c^ Solvent 1: 50% (*v/v*) DMSO in methanol. ^d^ SIF: simulated intestinal fluid. ^e^ Solvent 2: 10% (*v/v*) DMSO and 10% (*v/v*) formic acid in methanol. ^f^ BBMV: brush border membrane vesicle.

## Abbreviations

BBMV	brush border membrane vesicle
DMSO	dimethyl sulfoxide
FLA	flavonol-aglycone-rich fraction
FLG	flavonol-glycoside-rich fraction
HPLC	high-performance liquid chromatography
LPH	lactase-phlorizin hydrolase
PBS	phosphate-buffered saline
qPCR	quantitative polymerase chain reaction
qRT-PCR	quantitative real-time polymerase chain reaction
SCFA	short-chain fatty acid
SGF	simulated gastric fluid
SIF	simulated intestinal fluid
